# Action Myoclonus-Renal Failure Syndrome: A Case Report with Bioinformatic Annotations

**DOI:** 10.7759/cureus.41261

**Published:** 2023-07-01

**Authors:** Hakan Ekmekci, Omar Qutob, Huseyn Babayev, Ali Şahin

**Affiliations:** 1 Department of Neurology, Selcuk University Faculty of Medicine, Konya, TUR; 2 Department of Neurodevelopers, Silicosome Biotechnology, Konya, TUR; 3 Department of Microbiology, Abant Izzet Baysal University, Bolu, TUR

**Keywords:** scarb2 protein, bioinformatics, frameshift mutation, electromyography, action myoclonus-renal failure syndrome

## Abstract

Action myoclonus-renal failure (AMRF) syndrome is a rare autosomal recessive disorder characterized by myoclonic epilepsy with occasional renal failure comorbidity. This study examines a consanguineous family with multiple members presenting myoclonic epilepsy. The disease's continued transmission within the family is attributable to a lack of genetic testing and the inability to establish a definitive diagnosis. Our objective is to guide physicians toward accurate diagnoses and reduce the disease's recurrence through appropriate genetic counseling. Various diagnostic approaches can contribute to identifying AMRF. While magnetic resonance imaging (MRI) results and blood panels may not yield definitive diagnoses, electromyography (EMG) studies can serve as a robust diagnostic tool, leading to genetic confirmation. In line with standardized protocols, EMG findings consistent with AMRF present a polyneuropathy characterized by axonal degeneration and demyelinating features. These features manifest as decreased amplitude for axonal degeneration and decreased nerve conduction velocity (NCV) for demyelination. The presence of such EMG findings in a patient exhibiting both renal and central nervous system involvement may reinforce a preliminary diagnosis and warrant further genetic analysis.

## Introduction

Action myoclonus-renal failure (AMRF) syndrome encompasses a range of heterogeneous neurodegenerative conditions characterized by atypical seizures, poor response to treatment, and an overall unfavorable prognosis [1]. While existing case reports primarily emphasize the renal aspects of the disease, this report aims to highlight the progressive neurological components, which include, but are not limited to, myoclonus, dysarthria, generalized seizures, weakness, and preserved intellect [2]. Both renal and neurological manifestations of the condition are the result of a homozygous mutation in the SCARB2 gene and its associated protein, lysosomal integral membrane protein type 2 (LIMP-2). This mechanism, involving the accumulation of LIMP-2 in the endoplasmic reticulum (ER) and subsequent decrease in lysosomal activity, has also been observed in Gaucher's disease and Parkinson's disease [3,4]. LIMP-2 is a vital receptor that aids in the transportation of lysosomal hydrolases, including glucocerebrosidase (GBA), from the Golgi apparatus to the lysosome, thereby playing a crucial role in lysosomal function [5]. The involvement of SCARB2/LIMP-2 in normal autophagy suggests that its absence could result in the buildup of usually recycled proteins or organelles, leading to the formation of inclusions [6]. LIMP-2, a member of the CD36 scavenger receptor family member, is specifically involved in the transport of β-GBA from the ER to lysosomes [7].

## Case presentation

**Figure 1:**
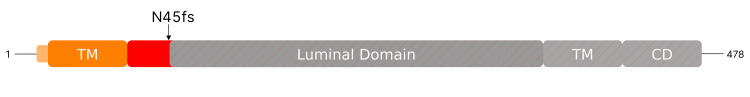
Schematic representation of the SCARB2 protein. The N45fs variant leads to the loss of functional parts of SCARB2, as indicated by the hatched areas. TM, transmembrane domain; CD, cytoplasmic domain

**Figure 2:**
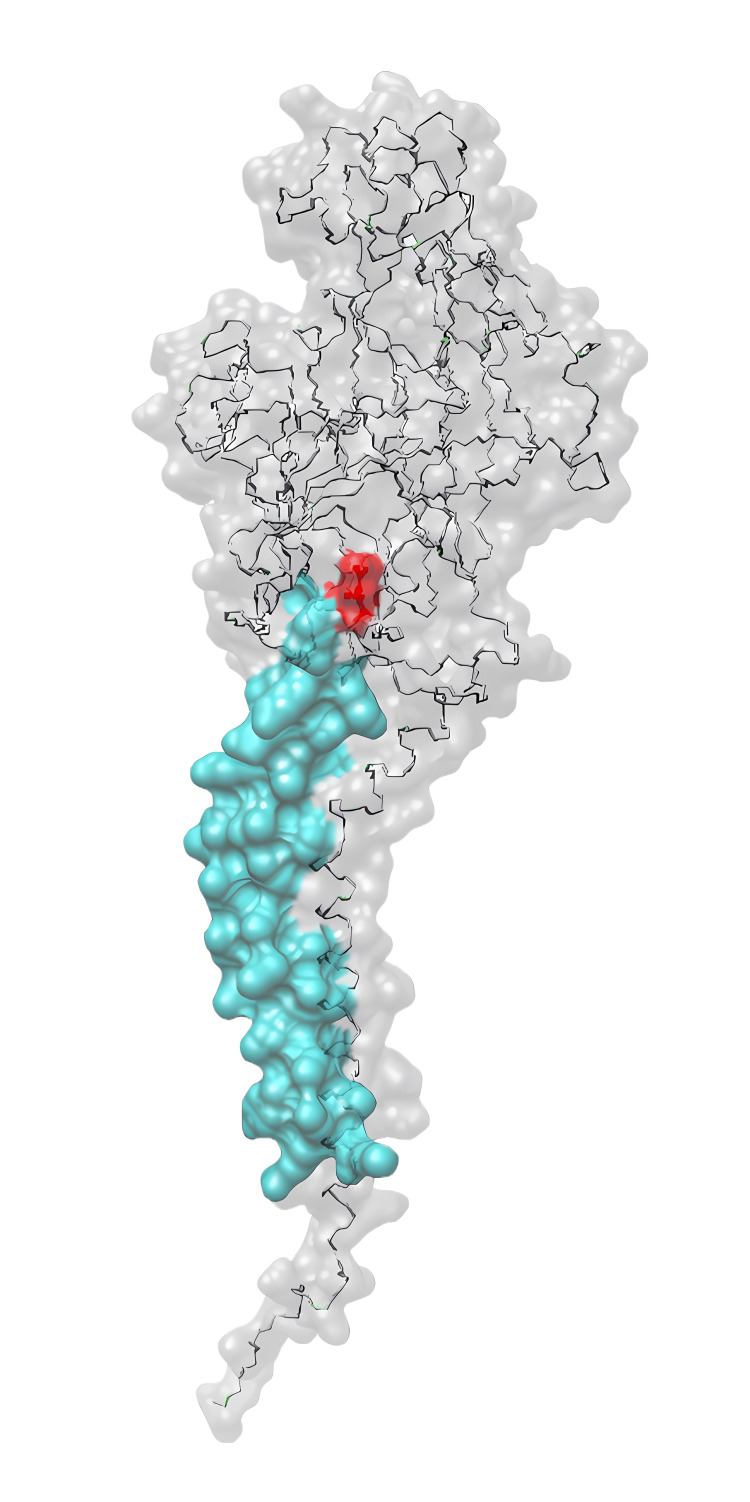
The modeled structure of SCARB2 and N45fs variant based on the AlphaFold model. The red color indicates the position of the amino acid mutation. Amino acids labeled with cyan represent the remaining part of SCARB2, while gray indicates the frameshift-affected region.

A 24-year-old female patient was diagnosed with epilepsy in 2020 due to progressive tremors and myoclonic episodes. She has since experienced three documented generalized myoclonic seizures, all occurring upon awakening from sleep and lasting around three minutes, followed by urinary incontinence. Additionally, she experienced constant myoclonic jerks, impacting her daily life. In 2021, a kidney biopsy was performed with a preliminary diagnosis of focal segmental glomerulonephritis (FSGN) due to proteinuria of approximately 2500 mg/24 hours. However, the results were inconclusive, and FSGN was excluded. The patient developed dysarthria after her diagnosis, prompting further medical evaluation and leading her to our neurological clinic, where she was subsequently hospitalized. The patient's parents are second-degree relatives. A detailed history revealed primary amenorrhea, with no menstruation without hormonal medications. Ultrasound examination showed the absence of ovarian tissue but the presence of the uterus and external female genitalia, indicating the need for karyotyping. The physical examination was unremarkable, except for pes cavus deformities. A neurological exam revealed cerebellar abnormalities, including dysmetria, dysdiadochokinesis, and ataxic gait. The patient had a hand myoclonus that developed while trying to grasp an object. A contrast MRI revealed no abnormalities. The presence of proteinuria, cerebellar abnormalities, myoclonic epileptic episodes, pes cavus, and EMG findings led to a broader differential diagnosis, with genetic conditions such as AMRF becoming the primary focus. Next-generation sequencing analysis in patients identified a homozygous SCARB2:c.104del variant in the SCARB2 gene. Bioinformatic studies determined the variant to be pathogenic. The SCARB2 gene was affected by a frameshift mutation affecting a significant portion of both the luminal and downstream domains (as shown in Figure 1). This led to the development of non-functional amino acid residues in the protein sequence, starting at amino acid 45, which is asparagine (N) in the wild-type protein. We created a 3D model of the mutant protein based on the structure of the LIMP-2 protein in the AlphaFold database [8,9] (Figure 2).

## Discussion

AMRF, characterized by renal and neurological involvement, is strongly reflected in the clinical presentation of most family members [10]. The phenotype typically manifests around the second decade of life and progressively worsens despite medical intervention [11]. Unlike other forms of hereditary epilepsy, intelligence is preserved in these patients, which is also a component of AMRF [10]. Various blood tests were conducted, with most parameters being normal. Remarkably, the patient's kidney function tests were also within normal ranges. Creatine was measured at 0.65 mg/dL and urea at 23 mg/dL, with an approximated eGFR of 136.17 mL/min/1.73 m^3^. Due to AMRF kidney involvement, an abnormal kidney function was expected. On the other hand, 24-hour urinary output studies showed around 1050.83 mg/day of proteinuria. Losartan was taken at a dosage of 50 mg once daily before the patient's admission to our neurology department. Although the medication has helped in reducing overall proteinuria, the patient consistently suffered hypotensive episodes as a side effect that significantly affected the quality of treatment. Following consultations with the nephrology department, it was determined that our approach to treatment should involve the consistent administration of medication while refraining from use during hypotensive periods. Other blood workups of note include sodium of 134 mEq/L, potassium of 3.05 mmol/L, chlorine of 94 mmol/L, and phosphorus of 5.0 mg/dL, while all other electrolytes measured were within normal ranges. Much like other case reports in the literature, magnetic resonance imaging (MRI) findings were normal regarding lack of pathology in both hemispheres, white and grey matter, basal ganglia, brain stem, and cerebellum [12]. The corpus callosum, pituitary gland, and optic nerve integrity were normal. Of specific note, there was no medial temporal, hippocampal, and parahippocampal gyrus asymmetry between the lobes; both lobes were considered of similar signal intensity on both T1 and T2 weighted images. Conducted studies include visual evoked potentials (VEP), brain stem auditory evoked potentials (BAEP), somatosensory evoked potentials (SEP), and electromyoneurography (ENMG) protocol. VEP, SEP, and BAEP studies produced normal results. However, ENMG results were much more revealing. Bilateral evaluations of median, ulnar, tibial, and popliteal nerves were performed. Upper extremity nerves showed decreased nerve conduction velocity (NCV) values, with the slowest conducting nerve in the upper extremity being the right median nerve at 40.8 m/s, indicating a demyelinating type of neuropathy. Lower extremity findings were even more pronounced, with NCV values going as low as 28 m/s in the left peroneal nerve. Table 1 includes motor nerve conduction study and Table 2 includes sensory nerve conduction study data.

**Table 1:** Data on electrophysiological examination of motor nerves.

Nerve	Site	Segment	Latency (ms)	Amplitude (mV)	NCV (m/s)
Median Nerve	Left	Wrsit	6.36	4.72	50.7
		Elbow	11.49	4.32	
	Right	Wrist	6.66	3.47	40.8
		Elbow	11.85	3.73	
Ulnar Nerve	Left	Wrist	4.95	4.57	-
		Below Elbow	9.15	3.54	57.1
		Above Elbow	11.3	3.43	53.5
	Right	Wrist	5.2	3.02	-
		Below Elbow	8.95	2.59	58.7
		Above Elbow	12.15	2.93	45.3
Peroneal Nerve	Left	Ankle	7.55	2.56	28.0
		Fibular Head	20.2	1.39	
	Right	Ankle	7.7	2.11	30.0
		Fibular Head	22.1	0.98	
Tibial Nerve	Left	Ankle	8.75	0.98	36.7
		Popliteal	20.75	1.06	
	Right	Ankle	10	0.87	38.7
		Popliteal	21.1	0.77	

**Table 2:** Data from electrophysiological examination of sensory nerves.

Nerve	Site	Segment	Latency (ms)	Amplitude (μV)	NCV (m/s)
Median	Right	Third Digit	4.8	3.50	29.2
Median	Left	Third Digit	5.44	1.50	28.5
Ulnar	Left	Fifth Digit	3.76	3.10	35.9

Furthermore, amplitudes also showed a decrease in all measured lower extremity nerves. The reduction in amplitude has traditionally been characterized as axonal neuropathy; nevertheless, upon considering the decline in NCV, the discernible pattern is indicative of a mixed sensory-motor polyneuropathy. These ENMG studies revealed a pattern of pathology featuring both demyelinating and axonal degeneration patterns. Such neuropathies are commonly seen in mitochondrial genetic conditions, Charcot-Marie-Tooth type II disease, and various hereditary polyneuropathies. With these differential diagnoses in consideration, previously attempted extensive genetic screening was broadened to include potential overlapping mutations and screen for previously mentioned conditions [2,3]. It is imperative to assess renal functionality in instances involving action myoclonus, and it is advisable to propose genetic diagnostic approaches for individuals exhibiting renal impairment associated with AMRF.

## Conclusions

AMRF is a rare, autosomal-recessive disease related to loss-of-function mutations in SCARB2 that encodes a LIMP-2. AMRF is characterized by progressive myoclonus epilepsy and renal failure. The report emphasizes that it is essential to conduct genetic testing for individuals who exhibit clinical signs and symptoms of AMRF, have a suspicion of having the condition, or have a family history of the disease. The study used next-generation sequencing technology to identify a new mutation in SCARB2. This finding can be beneficial for clinicians in diagnosing and treating patients with this uncommon condition.
